# An update on the diagnosis and management of the polyneuropathy of POEMS syndrome

**DOI:** 10.1007/s00415-018-9068-4

**Published:** 2018-09-27

**Authors:** Federica Cerri, Yuri Matteo Falzone, Nilo Riva, Angelo Quattrini

**Affiliations:** Neuropathology Unit, Division of Neuroscience and Department of Neurology, Institute of Experimental Neurology, San Raffaele Scientific Institute, Via Olgettina 48, 20132 Milan, Italy

**Keywords:** Vascular endothelial growth factor, Monoclonal gammopathy, CIDP, Nerve biopsy

## Abstract

POEMS syndrome is a rare, chronic, disabling paraneoplastic disorder characterized by peripheral neuropathy, organomegaly, endocrinopathy, monoclonal plasma cells disorder and skin changes. Diagnosis relies on the fulfillment of a set of clinical criteria of which polyneuropathy and a monoclonal plasma cell dyscrasia are early and essential features. Treatment may be either local or systemic and is aimed at the monoclonal plasma cell disorder. Our knowledge of the pathogenesis underlying the POEMS syndrome has advanced greatly over the past years, favoring an important progression in the recognition and management of this disorder. Here, we discuss the recent literature that has advanced our knowledge of the pathogenesis and clinical management of the polyneuropathy in POEMS syndrome.

## Introduction

POEMS (polyneuropathy, organomegaly, endocrinopathy, M-protein, and skin changes) syndrome is a rare multisystemic paraneoplastic disorder associated with osteosclerotic myeloma and increased serum and plasma levels of vascular endothelial growth factor (VEGF) [1–7]. Although an estimated median survival of 14 years has been reported [8], the presence and progression of several clinical features affecting the respiratory and nervous systems, among others, are potentially fatal if left untreated [8]. The diagnosis of POEMS syndrome relies primarily on the fulfillment of major and minor criteria [8, 9]. However, the peripheral neuropathy is almost always a constant feature [10–14]. Indeed, the identification of a chronic progressive, distal, sensorimotor polyneuropathy, along with a monoclonal plasma cell dyscrasia, is essential to the diagnosis of POEMS syndrome.

## Proposed disease mechanisms

Significant advances have been made in our understanding of the pathogenesis of POEMS syndrome, although a complete understanding of the underlying mechanisms has not yet been achieved. Current evidence supports the notion that at least some of its clinical findings, including peripheral neuropathy, are attributable to an increase in inflammatory cytokine levels rather than clonal plasma cells invasion [15].

VEGF is considered, together with other pro-inflammatory cytokines including tumor necrosis factor-alfa (TNF-α), interleukin-6 (IL-6) and interleukin-12 (IL-12), to be a relevant element in the pathogenesis of the disease [7, 16–21]. VEGF is a multifunctional cytokine, physiologically produced by osteoblasts and bone marrow-derived cells, including plasma cells [22–25]. It is pivotal in the regulation of angiogenesis and microvascular permeability by targeting different endothelial cell receptors [26]. Several upstream factors modulate VEGF production, such as the hypoxia-inducible transcription factor-1 (HIF-1), which is highly expressed in response to hypoxic conditions [20, 27]. Increased serum and plasma levels have been observed in the majority of cases and, therefore, considered a major criteria for the diagnosis of POEMS syndrome [4–7, 20, 21]. In addition, current evidence supports its value in the assessment of disease activity and treatment response [28–31]. The search of the origin of VEGF overproduction has led to controversial results. A recent study found higher levels of VEGF mRNA expression in the bone marrow plasma cells compared with CD138 negative cells [25]. Furthermore, polyclonal and monoclonal plasma cells showed comparable intracellular levels of VEGF, whereas monoclonal plasma cells exhibited higher levels of intracellular IL-6 expression [25], which is a known inducer of VEGF expression and secretion [5]. As mentioned previously, other inflammatory cytokines could be involved, since TNF-α and IL-6 were reported to be upregulated in the blood/serum/plasma of POEMS syndrome patients, whereas IL-12 showed significant correlation with disease activity and progression [16, 19, 32, 33].

Lenalidomide, a promising therapy for POEMS syndrome, appears to be effective particularly on oedema and peripheral neuropathy, probably due to its anti-VEGF effect [34–36]. In contrast, bevacizumab, an anti-VEGF antibody, gave ambiguous results with several reports of patients getting much worse after therapy [37–39]. This failure could be due to persistent high VEGF serum levels followed by a rapid decrease, leading first to endothelial cells hypertrophy and second to massive apoptosis inducing a capillary leak syndrome [40]. Alternatively, other angiogenic factors may play an important role in POEMS syndrome [41], justifying the limited clinical efficacy obtained by inhibition of VEGF alone. These controversial findings suggest that the interplay of several cytokines involved in angiogenesis and microvascular permeability, besides VEGF, might be significant in the pathogenesis of POEMS syndrome, at least by explaining some of its clinical features such as extra-vascular volume overload (ascites, pleural effusion and oedema), skin angioma, papilloedema and presumably peripheral neuropathy [7, 15, 17, 20]. Indeed, it is now accepted that VEGF is probably not the pathogenic initiating factor but a downstream mediator of a paraneoplastic syndrome.

## Multisystemic features and diagnosis of POEMS syndrome

POEMS is a rare syndrome with a broad spectrum of clinical presentations and laboratory features. In recent years, revised diagnostic criteria have been proposed and adapted considering the progress made in understanding this syndrome [8, 9]. The diagnosis of POEMS syndrome is confirmed when both the polyneuropathy and monoclonal gammopathy are present in association with one of the other three major criteria (Castleman disease, sclerotic bone lesions and increased levels of VEGF), and one of the six minor criteria (organomegaly, endocrinopathy, extravascular volume overload, skin changes, papilloedema, thrombocytosis/polycythemia).

Osteosclerotic lesions occur in approximately 95% of patients [42]. Computed tomography (CT) scans can show both densely sclerotic or lytic lesions with a sclerotic rim; mixed lesions with soap-bubble appearance have also been observed. Lesions are usually multiple and small in size, revealing avid FDG uptake as they enlarge [42]. Endocrinopathy occurs in approximately 84% of patients, with a high incidence of hypogonadism and hypopituitarism followed by thyroid abnormalities and adrenal insufficiency. As thyroid disease and diabetes mellitus are common in the general population, and the latter may be present in both patients with POEMS and chronic inflammatory demyelinating polyradiculoneuropathy (CIDP), they should not be considered alone as diagnostic criteria for POEMS syndrome. In such cases, a combination of endocrine abnormalities may be more reliable for the fulfillment of these criteria. Typically, organomegaly affects the liver, spleen, and lymph nodes, which can be easily detected on a CT scan. The skin changes are heterogenous, including hyperpigmentation, hypertrichosis, hemangioma, acrocyanosis, flushing, white nails and thickening [43, 44]. In two POEMS patient cohorts, papilloedema occurred in approximately 50% of cases during a 10 years follow-up period [45]. In one of these studies, the presence of papilloedema was identified as an independent adverse factor for overall survival [46]. Ischemic stroke is an uncommon but recognised feature reported in approximately 10% of POEMS patients [47, 48]. It has rarely been described as a first clinical manifestation, occurring approximately 23 months after symptoms onset on average [49]. Indeed, a thrombotic diathesis is present in POEMS syndrome, as witnessed by the presence of thrombocytosis, seen in approximately 55% of patients, and bone marrow plasmacytosis, leading to a higher risk of ischemic stroke [47]. POEMS-associated strokes tend to involve largely watershed zones, and only on a few cases is it due to cerebral large-vessel vasculitis. Extravascular volume overload generally manifests as peripheral oedema; ascites, pleural and pericardial effusion, pulmonary hypertension, renal dysfunction or cardiac insufficiency and may appear in advanced stages of the disease and is associated with a significantly shorter overall survival [8, 42].

Specific laboratory and recommended strategies for investigations are required to achieve the correct diagnosis and to guide adequate treatment in patients with POEMS syndrome. A thorough clinical assessment, including laboratory tests, neurophysiological and imaging studies, may be required to distinguish POEMS syndrome from other plasma cell dyscrasias and Castleman disease (CD). These tests include serum and urine protein electrophoresis and immunofixation, CT scans documenting osteoscleroic lesions, lymphadenopathy, organomegaly, ascites, pleural effusions, and oedema. Bone marrow aspirate and biopsy (test for kappa/lambda by immunohistochemistry) along with a serum and/or plasma VEGF level should be performed in case of suspected POEMS syndrome.

## Neuropathy in POEMS and mimic syndromes

The early diagnosis of POEMS syndrome is critical, as treatment options for the disease in more advanced stages are frequently unsuccessful, despite appropriate therapy being applied. Delays in diagnosis are common and this has been reported to be higher than 13 months from symptoms onset in many cases [8]. This is due to the challenges in distinguishing POEMS syndrome at the onset from acquired chronic CIDP [14, 50–52]. Furthermore, POEMS syndrome should be distinguished from other neuropathies associated with monoclonal gammopathies such as monoclonal gammopathy of undetermined significance (MGUS) [14, 53, 54]. Although B-cell disorders are usually non-malignant, peripheral neuropathy also occurs with multiple myeloma, Waldenström’s macroglobulinemia (WM), lymphoma, primary amyloidosis (AL), and lymphocytic leukemia [55, 56]. Correct diagnosis of the above polyneuropathies is essential, as different disorders respond to different treatments [57, 58].

Peripheral neuropathy is frequently the presenting clinical feature, and it is commonly described as a progressive ascending, symmetric sensorimotor demyelinating polyneuropathy, sometimes evolving into polyradiculoneuropathy. Typically, patients show a high stepping gait. Prominent pain in the legs is usually reported in patients with POEMS, compared with patients with CIDP [59]. With the progression of the disorder, severe distal weakness at both lower and upper limbs ensues [8, 14]. Although damage in both POEMS and CIDP is primarily demyelinating, neurophysiological studies in patients with POEMS show a higher degree of length-dependent axonal loss, especially in the lower limbs, as evidenced by reduced amplitudes of compound muscle action potentials and more fibrillation potentials compared with CIDP [60, 61]. Furthermore, a more prominent slowing of nerve conduction in the intermediate than in the distal nerve segments has been reported, while demyelination in CIDP shows multifocal pattern involving both distal and intermediate segments [62]. Conduction blocks and temporal dispersion are less frequent in POEMS patients than CIDP. Cerebrospinal fluid examination and nerve imaging may show abnormalities but these are not specific for POEMS syndrome and can also be seen in CIDP, such as elevated cerebrospinal fluid protein levels and increased enhancement and enlargement of nerve roots [63]. Diabetes or glucose intolerance have been associated with both conditions.

The presence of a λ-type IgA or IgG monoclonal gammopathy is mandatory for the diagnosis of the POEMS syndrome. In MGUS-associated neuropathy the paraprotein is more commonly IgM [64–67], and the associated neuropathies are heterogenous, showing demyelinating, axonal, or axonal/demyelinating types [65–67]. In about half the patients with an IgM M-protein, the monoclonal protein reacts with myelin-associated glycoprotein (anti-MAG antibodies) [68–70], leading to a distal acquired demyelinating neuropathy (DADS). Patients with anti-MAG antibodies typically present with a chronic and slowly progressive, distal, symmetric, predominantly sensory demyelinating polyneuropathy, which begins with numbness and paresthesia in feet or hands and slowly progresses [69–71]. A deep sensory involvement is present, characterized by ataxia, loss of joint position, Romberg’s sign and postural tremor in the upper limbs. However, multisystemic involvement is not observed in DADS and the disease is usually limited to the distal segments of the limbs. Besides DADS, an IgM isotype is also associated with WM, a malignant lymphoproliferative disease characterized by the clonal expansion of B-cells [72]. Although WM may share neuropathy and papilloedema with POEMS syndrome [73], a bone marrow biopsy is effective in distinguishing the two disorders [72, 74]. Neuropathies associated with IgG and IgA gammopathies are less common than neuropathies associated with IgM and are even more heterogeneous [64, 75]. Patients affected by IgG and IgA gammopathies present with features of demyelinating, axonal, or mixed neuropathies.

CD is a rare lymphoproliferative disorder, characterized by enlarged lymph nodes, and occurs in approximately 11–30% patients with POEMS syndrome [76]. CD can be classified according to whether the lymphadenopathy is unicentric or multicentric. Peripheral neuropathy is present in approximately 25% of patients with multicentric CD [14, 77, 78]. Unlike POEMS syndrome, CD patients present with a painless distal sensory demyelinating neuropathy, which is often mild in severity [14, 79]. Although the pathogenesis of CD is unclear, VEGF, IL-6, and IL-1 may play an important role in the development of the disease. However, in contrast to POEMS, the predominantly elevated cytokine is IL-6, and not VEGF [14].

AL is a multisystemic disease characterized by widespread amyloid deposition, and diagnosis implies the presence of plasma cell dyscrasia [80], commonly lambda-restricted monoclonal plasma cells. Peripheral neuropathy is a common manifestation of AL, with distal sensory symptoms, including painful dysesthesia, loss of light touch and temperature sensation, and marked autonomic involvement being the main clinical feature [81, 82]. Distal symmetric weakness develops during the progression of AL. Patients with AL have electrophysiological features consistent with an axonal sensorimotor polyneuropathy, frequently associated with an autonomic neuropathy and carpal tunnel syndrome [83]. The diagnosis relies on the demonstration of amyloid deposits in tissue obtained from involved organs [82]. Bone marrow findings and the plasma or serum VEGF level are fundamental to distinguish POEMS syndrome from AL [9, 29].

## Pathological studies

Sural nerve biopsy is rarely necessary to make the diagnosis of POEMS syndrome. However, nerve biopsy could be a useful tool in distinguishing POEMS syndrome from AL. In addition, pathological studies can contribute significantly to understanding the pathogenic mechanisms of nerve damage in POEMS syndrome [7, 20, 84].

**Fig. 1:**
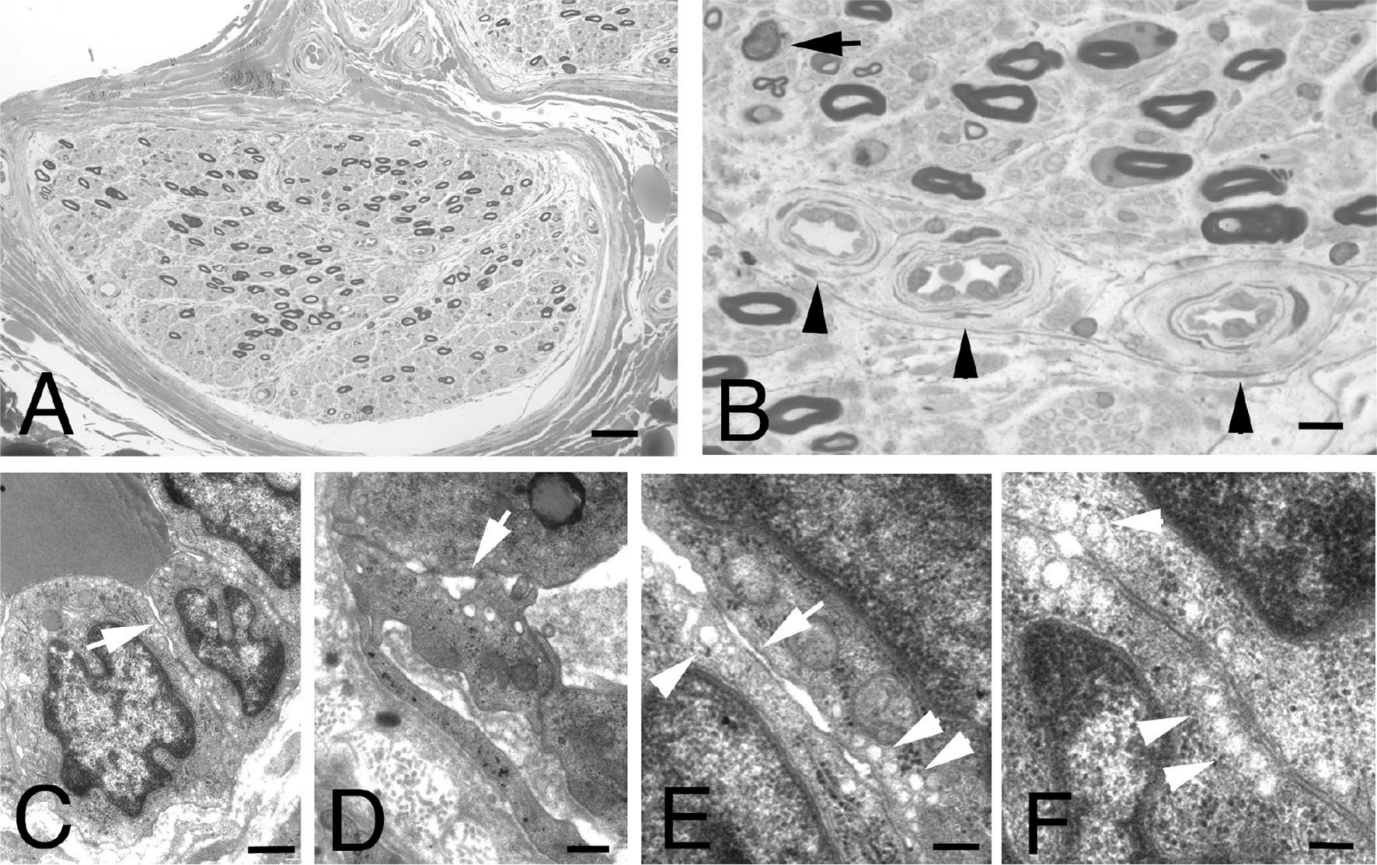
Transverse semi thin sections of a sural nerve from a patient with POEMS syndrome showing diffuse reduction of the myelinated nerve fibers (**a**), axonal degeneration (**b**, arrow) and thickening of the vessel walls (**b**, arrowhead) due to basal lamina and cellular proliferation. Electron micrographs showing endothelial cytoplasmic enlargement (**c**) and gap between endothelial cells (**c**–**e**, arrows). Many pinocytic vesicles adjacent to the cell membranes are present (**e** and **f**, arrowhead). Scale bars: **a**: 40 µm; **b**: 10 µm; **c**: 2 µm; **d**–**f**: 500 nm

Nerve biopsy in POEMS syndrome reveals major signs of demyelination with uncompacted myelin on electron microscopy in the absence of macrophage-associated demyelination [84–87], as observed in CIDP [88]. However, in our experience, nerve pathology in POEMS syndrome may also show predominant axonal damage characterized by loss of myelinated nerve fibers and the presence of myelin ovoids (Fig. 1), indicating acute axonal degeneration [20]. In addition, ultrastructural analysis of POEMS nerves has revealed endothelial cytoplasmic enlargement, opening of the tight junctions between endothelial cells, and presence of many pinocytic vesicles adjacent to the cell membranes (Fig. 1), suggesting an alteration in permeability of endoneurial vessels. Furthermore, the vascular damage correlated with the VEGF serum levels in patients. Thus, we propose that the mechanism underlying the peripheral neuropathy in POEMS syndrome is due to endothelial injury, indirectly or directly caused by an abnormal activation of endothelial cells by VEGF, which is overexpressed in the nerves of patients with POEMS syndrome, thereby inducing microvascular changes and impaired vascular permeability [7, 17, 20, 89]. These alterations lead to a secondary ischemic microangiopathy and chronic axonal damage of nerve fibers through alteration of the blood–nerve barrier [20]. Finally, the disruption of vascular and metabolic dynamics in the nerve leads to a perpetuating hypoxic state, where secondary increases in local VEGF occurs due to induced HIF-1a expression, fueling a positive destructive feedback whereby damage progresses relentlessly. Similarly, vessel structural derangement was also reported in skin capillaries [90].

**Fig. 2:**
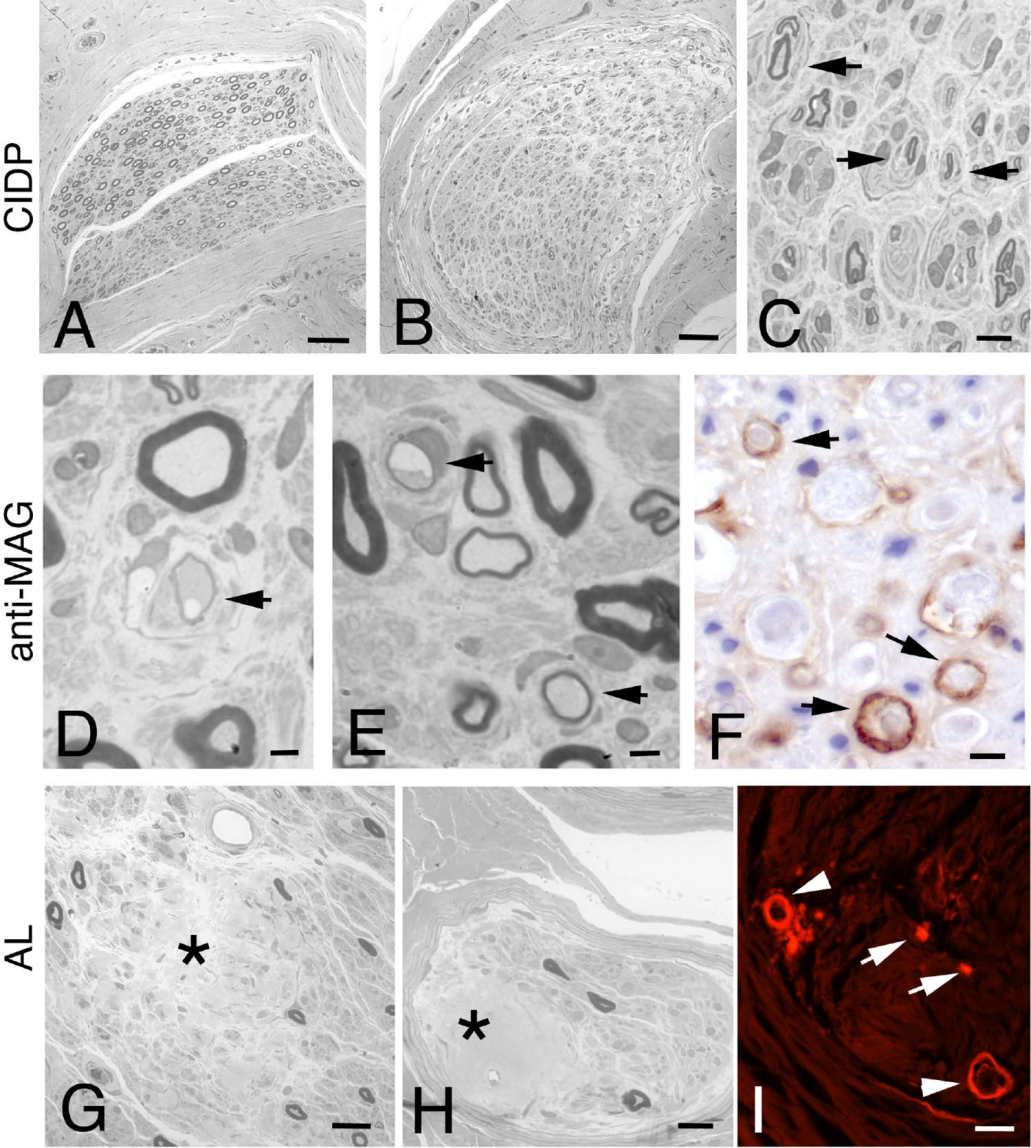
Representative photomicrographs illustrating the nerve biopsy from patients with CIDP (**a**–**c**), anti-MAG (**d**–**f**) and amyloid neuropathy (AL) (**g**–**i**). In CIDP nerves, different degrees of large myelinated fibers loss between fascicles is evident (**a** and **b**); one fascicle has greater myelinated fibers loss (**b**), showing remyelination and multiple large onion bulbs (**c**, arrows). Demyelination and onion bulb formation in anti-MAG neuropathy (**d** and **e**, arrows); direct immunohistochemistry shows localization of IgM to the myelin sheath (**f**, arrows). Nerve biopsy of patient with AL shows severe reduction of myelinated nerve fibers and diffuse amyloid deposits in the endoneurium (**g**, asterisk) and in perivascular space (**h**, asterisk); histological slides stained with Congo red under under fluorescent microscopy showing scattered amyloid deposits in the endoneurium (**i**, arrows) and perivascular region (**i**, arrowhead). Scale bars: **a** and **b**: 60 µm; **c**: 10 µm; **d**–**f**: 5 µm; **g** and **h**: 20 µm; **i**: 40 µm

Pathological evidence indicates that CIDP is caused by an inflammatory demyelination of spinal roots and peripheral nerves. Macrophages play a fundamental role in CIDP. The pathological hallmark of CIDP is macrophage-mediated segmental demyelination [88]. Nerve biopsies showed endoneurial edema and endoneurial perivascular infiltrates composed of macrophages and T lymphocytes [91, 92]. Inflammatory lymphocytes have been observed in the perineurium, whereas in POEMS inflammatory cells are scarce and localized perivascularly in the epineurium when present. In CIDP, progression of the disorder, associated with repeated demyelination and remyelination processes, results in onion bulb formation and axonal degeneration [93]. In addition, the pattern of damage in CIDP, including the fiber loss, follows a multifocal rather than diffuse pattern (Fig. 2) [94, 95], and this represents an important factor in distinguishing CIDP from other demyelinating polyneuropathies such as that seen in the POEMS syndrome [95].

Pathological studies in neuropathy with anti-MAG antibodies reveals demyelination (Fig. 2) in association with widening of myelin lamellae on electron microscopic examination [96] along with deposits of IgM and complement on myelin sheaths as demonstrated by direct immunohistochemistry (Fig. 2) [97, 98]. Immunocytochemical studies using anti-MAG antibodies from these patients has revealed that the antibodies were bound to the myelin. In nerve from patients with WM, pathological findings are similar to those seen in the neuropathy associated with anti-MAG [99]. However, in many patients with WM, axonal damage is prevalent due to a different mechanism of nerve damage, including vasculitic neuropathy, amyloid light chain deposition and tumoral infiltration of the nerve. Thus, nerve biopsy should be considered in patients showing a rapidly evolving course associated with pain and weight loss in WM. Neuropathies associated with IgG and IgA gammopathies are less common and present with features of a demyelinating, axonal, or mixed neuropathy.

The diagnosis of amyloid neuropathy depends on the demonstration of amyloid deposits in nerve biopsies [82]. Congo red staining can show amyloid deposits in the epineurium, perineurium or endoneurium (Fig. 2). In the endoneurium, amyloid can be observed focally in the vessel wall or with a large diffuse distribution in the endoneurial interstitial compartment. In our experience, in AL, amyloid deposits are prominent in and around vessel walls, while more diffuse deposits in the endoneurium are present in hereditary amyloid neuropathies. In AL, the fundamental process is axonal degeneration. Although in early stages of the disease a selective loss of unmyelinated and small myelinated fibers is usually present, all fibers become severely affected with the progression of the disorder (Fig. 2).

## Treatment and clinical management

An early and prompt diagnosis is fundamental to provide the best therapeutic intervention and follow-up recommendations. A multidisciplinary approach is essential for patients with POEMS syndrome and requires a core team of healthcare providers including a hematologist, neurologist, neurophysiologist and physiotherapist.

Treatment of POEMS syndrome is directed at the underlying monoclonal plasma cell disorder and depends on the extent of the disease and bone marrow involvement. In patients with two or fewer plasmacytoma lesions, radiotherapy is the first-line therapy. Patients with generalised disease, characterized by diffuse sclerotic lesions or disseminated bone marrow involvement, should be treated with systemic therapy [9, 40]. Notably, plasmapheresis and intravenous immunoglobulin used in immune-mediated neuropathies seem unsuccessful [100]. High-dose chemotherapy-conditioned autologous stem cell transplantation is the current gold standard treatment for POEMS syndrome, showing good hematological control, neurological response, with improvement of the neuropathy, and good survival [101–104]. However, patients in advanced stage of disease cannot undergo autologous stem cell transplantation.

Potential new therapeutic approaches involve targeting key factors in the pathogenesis of POEMS, including VEGF, IL-6, IL-12 and TNF [40]. Thalidomide and lenalidomide (a derivative of thalidomide) are candidates to treat patients at high risk for transplantation because of their effectiveness against plasma cell proliferation, anti-VEGF and anti-cytokines effect [105]. Although thalidomide can strongly inhibit VEGF production, it can induce a toxic neuropathy and requires careful monitoring. Lenalidomide has a much lower risk of a peripheral neuropathy and has been shown to be effective against neuropathy and edema, reducing the serum levels of VEGF [34–36]. Indeed, treatment with lenalidomide in POEMS showed an improvement of the neuropathy in 90% of patients.

Neurorehabilitation is essential in patients with sensory and motor impairment. Orthopedic shoes to prevent foot-drop, ankle–foot orthotics and other supporting devices can be used in patients with a debilitating neuropathy to improve quality of life. Pain may be a dominant feature in patients with POEMS and of course should be treated pharmacologically with drugs such as gabapentin and tricyclic antidepressants.

## Conclusions

In summary, important progress has been made in the diagnosis and management of POEMS syndrome since its first description. Measurement of VEGF levels in the serum and plasma may help to make a rapid diagnosis, which is essential for the treatment of patients before severe neurologic impairment ensues. Early diagnosis and therapy can prevent progression and irreversible secondary axonal degeneration. Advances in our understanding of the underlying pathology in POEMS syndrome has identified new targets for future therapeutic efforts, particularly VEGF. However, more research is needed to understand what causes the excess production of cytokines that leads to the multiple clinical manifestations, and whether genetic factors are implicated in POEMS syndrome.
